# Optimal Selection of Biodegradable Polymer Composites for Load-Bearing Bone Tissue Engineering: A Hybrid Fuzzy AHP-TOPSIS Framework with Sensitivity-Based Robustness Analysis

**DOI:** 10.3390/jfb17050258

**Published:** 2026-05-21

**Authors:** Lafi Hamidat, Dilber Uzun Ozsahin, Berna Uzun

**Affiliations:** 1 Department of Biomedical Engineering, Near East University, TRNC, Mersin 10, Nicosia 99138, Turkey; 2 Department of Medical Diagnostic Imaging, College of Health Sciences, University of Sharjah, Sharjah P.O. Box 27272, United Arab Emirates; 3 Research Institute for Medical and Health Sciences, University of Sharjah, Sharjah P.O. Box 27272, United Arab Emirates; 4 Irfan Suat Gunsel Operational Research Institute, TRNC, Mersin 10, Nicosia 99138, Turkey

**Keywords:** bone tissue engineering, biodegradable composites, fuzzy AHP-TOPSIS, material selection, PLA/Hydroxyapatite, triangular fuzzy numbers

## Abstract

The development of biodegradable scaffolds for load-bearing bone tissue engineering (BTE) presents a fundamental multi-criteria optimization challenge, requiring a simultaneous balance among mechanical performance, biological integration, and degradation kinetics. These criteria are inherently conflicting: composite formulations with the highest compressive strength frequently exhibit suboptimal porosity, while those with superior osteoconductivity often lack sufficient load-bearing capacity. To address this challenge rigorously, this study establishes a hybrid Fuzzy Analytic Hierarchy Process–Technique for Order of Preference by Similarity to Ideal Solution (Fuzzy AHP-TOPSIS) framework to evaluate and rank five clinically relevant biodegradable polymer–ceramic composite candidates: PLA/Hydroxyapatite (PLA/HA), PCL/Hydroxyapatite (PCL/HA), PLGA/Bioactive Glass (PLGA/BG), PLA/Carbon Nanotubes (PLA/CNT), and PLA/Magnesium (PLA/Mg). Quantitative property data were systematically extracted from ten peer-reviewed experimental studies published between 2021 and 2025, and converted into Triangular Fuzzy Numbers (TFNs) to explicitly model inter-study variability arising from differences in fabrication methods, filler loading, and testing conditions. Fuzzy AHP analysis identified Compressive Strength (w = 25.2%) and Cell Viability (w = 21.5%) as the dominant decision criteria for load-bearing cortical bone repair. The Fuzzy TOPSIS ranking identified PLA/HA as the optimal composite candidate (Closeness Coefficient, CCᵢ = 0.677), demonstrating the superior multi-criteria balance required for cortical bone repair applications. Although PLA/CNT achieved the highest mechanical strength, it was outranked due to lower osteoconductivity and elevated cytotoxicity uncertainty at high nanotube concentrations (CCᵢ = 0.544). Sensitivity analysis across five distinct weighting scenarios confirmed the robustness of PLA/HA as the primary candidate. These findings provide a validated, replicable computational blueprint for evidence-based scaffold material selection, with direct implications for reducing the burden of costly trial-and-error experimentation in BTE research.

## 1. Introduction

The repair of critical-sized bone defects defined as those that will not spontaneously heal without surgical intervention remains one of the most pressing challenges in contemporary orthopaedic and maxillofacial surgery, particularly for load-bearing sites such as segmental defects of the femur or tibia [1]. Approximately 2.2 million bone graft procedures are performed globally each year, and the associated healthcare burden is expected to intensify as the global population ages [2]. While autologous bone grafting remains the clinical reference standard by virtue of its osteoinductive, osteoconductive, and osteogenic properties, its clinical utility is constrained by donor-site morbidity, limited graft volume, prolonged operative time, and significant post-operative pain [2]. Allograft alternatives circumvent supply limitations but introduce risks of immune rejection, disease transmission, and compromised biological activity following sterilization [3]. These limitations have catalyzed intensive research into synthetic biodegradable scaffolds, which aim to provide a temporary, load-bearing three-dimensional template for cell attachment, proliferation, and extracellular matrix deposition, before gradually degrading in situ and being replaced by native regenerated tissue [3,4].

The material selection process for load-bearing BTE scaffolds is characterized by a demanding and often irreconcilable set of design requirements. Mechanically, the scaffold must possess compressive strength and elastic modulus values sufficiently close to those of cortical bone (100–150 MPa and 12–18 GPa, respectively) to prevent stress shielding at the implant interface and avoid mechanical collapse during the early remodeling phase [5]. Biologically, a minimum porosity of 50–60% with an interconnected pore architecture (≥300 μm) is required to facilitate vascularization, nutrient transport, and cellular ingrowth [6,7,8]. Temporally, the scaffold’s degradation kinetics must be synchronized with the rate of new bone formation, typically 3–6 months for cortical bone repair, to maintain structural integrity during the critical early healing window while avoiding the persistence of a foreign body [5,6,9,10].

Synthetic biopolymers, including Polylactic Acid (PLA) [11], Polycaprolactone (PCL) [12], and Poly(lactic-co-glycolic acid) (PLGA), are widely employed in BTE owing to their established biodegradability, processability, and regulatory approval status. However, in isolation, these polymers exhibit mechanical properties far inferior to cortical bone and lack inherent osteoconductivity. Bioceramics such as Hydroxyapatite (HA) and Bioactive Glass (BG) offer excellent bioactivity and chemical affinity with native bone mineral, but are intrinsically brittle and prone to significant performance limitations under tensile or shear loading [13]. To overcome these individual limitations, polymer–matrix composites (PMCs) have emerged as the predominant research strategy: by incorporating ceramic, metallic, or carbonaceous fillers into a biopolymer matrix, it becomes possible to engineer a material whose mechanical and biological profile is greater than the sum of its components [14,15].

Despite the substantial volume of experimental literature on PMC scaffolds, there is a conspicuous absence of a standardized, quantitative framework for systematically comparing candidate formulations. The vast majority of published reviews rely on qualitative descriptive analysis, rendering objective identification of the “optimal” composite for a specific clinical context both difficult and subjective [16]. Furthermore, material property values reported across independent studies vary considerably due to differences in fabrication methodology (e.g., 3D printing versus solvent casting), filler loading fraction, and mechanical testing protocols variability that crisp-value comparison methods discard rather than model [17].

This study addresses these limitations by applying a hybrid Multi-Criteria Decision-Making (MCDM) framework that integrates Fuzzy Set Theory with the Analytic Hierarchy Process (AHP) and TOPSIS. The Fuzzy AHP component provides a structured, expert-informed method for deriving criterion weights that reflects the relative clinical importance of each design requirement. The Fuzzy TOPSIS component generates a complete, quantitative ranking of all candidate composites. Critically, the integration of Triangular Fuzzy Numbers (TFNs) throughout the framework preserves and propagates the inter-study variability inherent in literature-sourced data, enabling a more clinically conservative and biologically cautious ranking than crisp-value alternatives. A sensitivity analysis across five distinct expert weighting scenarios further validates the robustness of the final selection. This “in silico” approach provides a statistically grounded, replicable decision blueprint that can substantively reduce the reliance on exhaustive physical experimentation in BTE research.

## 2. Materials and Methods

### 2.1. Research Framework

**Figure 1:**
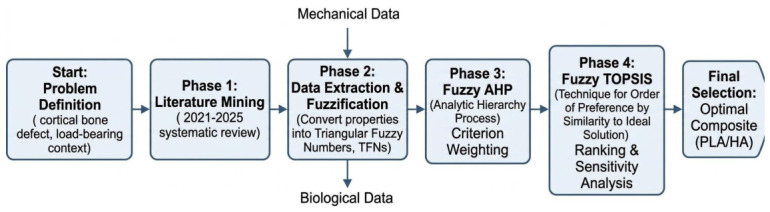
Flowchart of the four-phase research methodology. Phase 1: Problem definition and literature mining; Phase 2: Data extraction and fuzzification; Phase 3: Fuzzy AHP criterion weighting; Phase 4: Fuzzy TOPSIS ranking and sensitivity analysis.

The study was conducted across four sequential, methodologically integrated phases: (1) systematic identification and hierarchical structuring of evaluation criteria; (2) systematic data acquisition from peer-reviewed experimental literature (2021–2025) and conversion of raw property intervals into Triangular Fuzzy Numbers; (3) determination of criterion weights via Fuzzy AHP; and (4) ranking of composite candidates via Fuzzy TOPSIS, followed by sensitivity analysis. The overall workflow is illustrated in Figure 1.

### 2.2. Systematic Data Acquisition

To ensure scientific rigor and reproducibility, all quantitative material property data were extracted from primary peer-reviewed experimental studies rather than generic material datasheets. A systematic literature search was conducted across three bibliographic databases: Scopus, Web of Science, and PubMed, using the structured Boolean search syntax: (“Polymer Composite” OR “Scaffold”) AND (“Bone Tissue Engineering”) AND (“Mechanical Properties” OR “Compressive Strength”). The search was restricted to studies published between January 2021 and December 2025 to ensure that the data matrix reflects the current state of the art. The full inclusion and exclusion criteria applied during study selection are summarized in Table 1.

**Figure 2:**
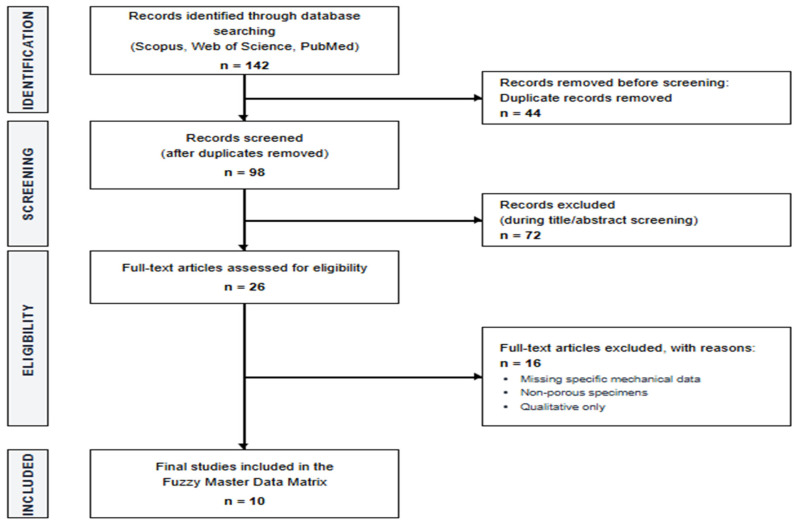
Prisma Flow Chart.

The systematic review followed the PRISMA (Preferred Reporting Items for Systematic Reviews and Meta-Analyses) guidelines as seen in Figure 2 [18]. To ensure data integrity, the extraction of mechanical and biological properties was performed independently by two researchers (I.I.S. and L.H.). Any discrepancies in reported property ranges were resolved through consensus or via consultation with a third senior expert (D.U.O.). This dual-reviewer protocol was implemented to minimize selection bias and ensure the accurate conversion of experimental intervals into Triangular Fuzzy Numbers.

### 2.3. Fuzzy Logic Implementation and Justification

Experimental data in bone tissue engineering research exhibits substantial variability arising from differences in 3D printing parameters, filler loading fractions, sintering temperatures, and mechanical testing protocols [19]. Reporting a single crisp value (e.g., the midpoint of a reported range) would both discard meaningful information regarding the distribution of plausible values and fail to propagate this uncertainty into the final ranking. To address this, all quantitative property intervals [a, b] and qualitative expert assessments were converted into Triangular Fuzzy Numbers (TFNs), denoted Ã = (l, m, u), where l is the lower bound (most pessimistic value), m the modal (most likely) value, and u the upper bound (most optimistic value) [20]. For a quantitative interval [a, b], this yields the TFN (a, (a + b)/2, b). The linguistic scale employed for expert judgements in the AHP pairwise comparison matrices is defined in Table 2.

### 2.4. Composite Candidates and Selection Justification

Five composite candidates were selected to represent the principal material strategies currently under investigation in load bearing BTE research. The selection was guided by citation frequency in recent systematic reviews, diversity of filler mechanism (ceramic, carbonaceous, metallic), and clinical translation potential. The candidates, their component roles, and justification for inclusion are presented in Table 3.

### 2.5. Fuzzy Master Data Matrix

Quantitative property data for all five composites were extracted from the ten included studies and processed into the Fuzzy Master Data Matrix presented in Table 4. Six evaluation criteria were defined across two domains: mechanical performance (Compressive Strength, C1; Elastic Modulus, C2; Porosity, C3; Degradation Rate, C4) and biological/economic performance (Cell Viability, C5; Manufacturing Cost Index, C6). For each criterion, the TFN (l, m, u) was derived from the reported range of values across the included studies for that composite.

**Figure 3:**
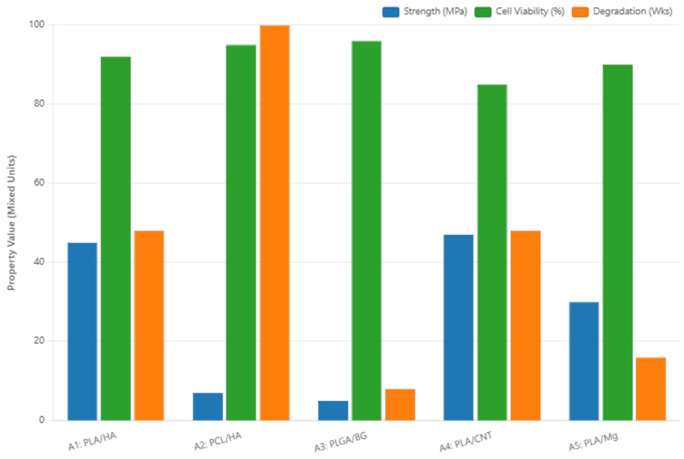
Conflict analysis of composite properties: comparison of modal values for compressive strength (MPa), cell viability (%), and degradation rate (weeks, normalized) across the five candidate composites. The divergence between mechanical and biological performance axes illustrates the fundamental trade-off that the Fuzzy AHP-TOPSIS framework is designed to resolve.

A visual comparison of the modal property values across the five composites, highlighting the trade-offs between mechanical strength, cell viability, and degradation rates, is presented in Figure 3.

### 2.6. Fuzzy AHP: Criterion Weighting Procedure

**Figure 4:**
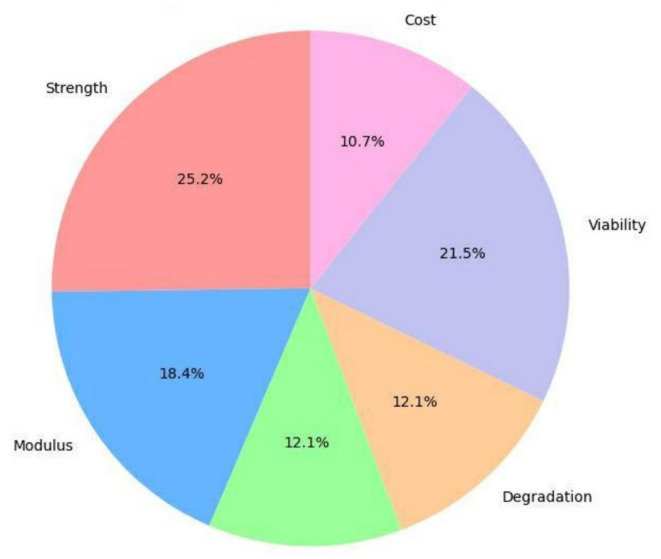
Distribution of Fuzzy AHP-derived global criterion weights. Compressive Strength (C1, 25.2%) and Cell Viability (C5, 21.5%) collectively account for 46.7% of total weight, reflecting the expert consensus that mechanical load-bearing capacity and biological safety are co-primary requirements for cortical bone scaffold applications.

An expert panel comprising two senior biomedical engineers with specialization in scaffold fabrication and an orthopaedic surgeon experienced in bone defect reconstruction employed pairwise comparison matrices to assign relative importance weights to the six evaluation criteria. Pairwise comparisons were made using the TFN linguistic scale defined in Table 2, following the Chang extent analysis method for Fuzzy AHP [20]. The geometric mean of the expert assessments was computed to consolidate panel judgements into a single fuzzy comparison matrix. Consistency ratios were calculated following defuzzification of the crisp matrix to ensure panel consistency (CR < 0.10 for all matrices) [20]. The resulting global criterion weights are presented in Table 5 and Figure 4.

### 2.7. Fuzzy TOPSIS Ranking Procedure

Fuzzy TOPSIS was implemented in four steps following the method of Chen (2000) [17]: (i) construction of the weighted normalized fuzzy decision matrix by multiplying each normalized TFN performance value by the corresponding fuzzy criterion weight from Table 5; (ii) identification of the Fuzzy Positive Ideal Solution (FPIS, A*) and Fuzzy Negative Ideal Solution (FNIS, A^−^) as the maximum and minimum performance TFN values across all alternatives for each criterion, respectively; (iii) calculation of the distance of each alternative from FPIS (dᵢ*) and FNIS (dᵢ^−^) using the vertex distance formula for triangular fuzzy numbers; and (iv) computation of the Closeness Coefficient CCᵢ = dᵢ^−^/(dᵢ* + dᵢ^−^) for each alternative, where CCᵢ ∈ [0, 1] and a higher value indicates closer proximity to the ideal solution. Degradation Rate (C4) was treated as a benefit criterion with a target range of 12–26 weeks; Cost (C6) was treated as a cost criterion.

## 3. Results

### 3.1. Fuzzy AHP Criterion Weights

The Fuzzy AHP analysis determined weights consistent with the biomechanical requirements of cortical bone repair. Compressive Strength (C1) received the highest weight (25.2%), followed by Cell Viability (C5, 21.5%) and Elastic Modulus (C2, 18.4%). Together, these three criteria account for 65.1% of total decision weight, reflecting the expert consensus that a load-bearing scaffold must simultaneously achieve structural competence and biological safety as co-primary requirements. Porosity (C3) and Degradation Rate (C4) received equal intermediate weights (12.1% each), acknowledging their importance for vascularization and temporal remodeling synchrony. Manufacturing Cost (C6) was deliberately assigned the lowest priority (10.7%), consistent with the established view in the BTE literature that clinical performance considerations should dominate at the material selection stage, with cost optimization addressed at the scale-up and manufacturing stage [29].

### 3.2. Fuzzy TOPSIS Ranking

**Figure 5:**
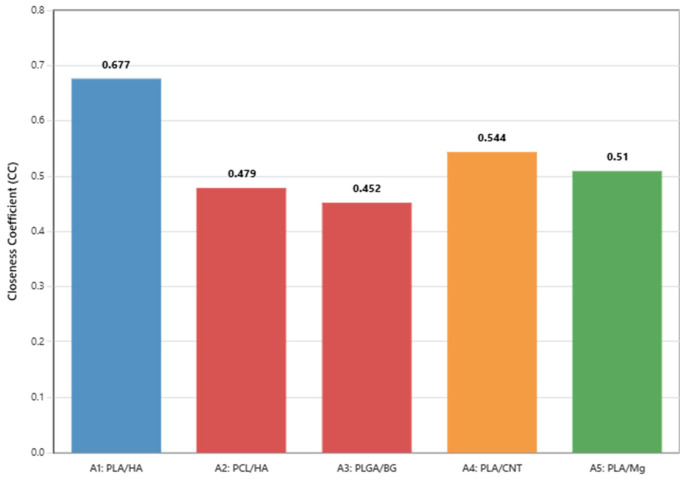
Final Fuzzy TOPSIS Closeness Coefficient (CCᵢ) scores for all five biodegradable composite candidates. PLA/HA (A1) achieves the highest score (CCᵢ = 0.677), outperforming the nearest competitor, PLA/CNT (A4), by a margin of 0.133.

The Fuzzy TOPSIS algorithm computed the closeness coefficient for each composite candidate. The complete ranking is presented in Table 6, and the CCᵢ scores are illustrated in Figure 5.

PLA/HA (A1) emerged as the most favorable candidate under the current modeling assumptions composite, achieving CCᵢ = 0.677 and outperforming the nearest competitor (PLA/CNT) by a margin of ΔCCᵢ = 0.133. This gap reflects not the exceptional performance of PLA/HA on any single criterion, but its uniquely consistent multi-criteria profile: it avoids the critical biological failure of PLA/CNT (cytotoxicity uncertainty), the mechanical failure of PCL/HA (yield strength < 1 MPa), and the temporal failure of PLGA/BG (degradation window of only 4–12 weeks). The complete ranking with CCᵢ values is depicted in Figure 5.

### 3.3. Sensitivity Analysis

**Figure 6:**
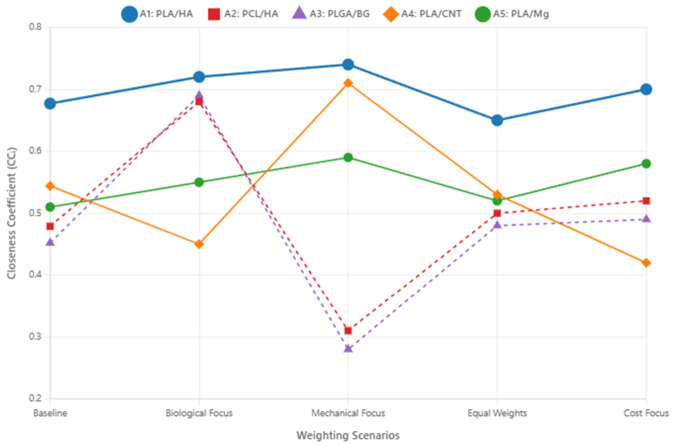
Sensitivity analysis: Closeness Coefficient (CCᵢ) for each composite candidate across five expert weighting scenarios. PLA/HA (A1) maintains the highest CCᵢ value across all five scenarios, confirming the robustness of its top ranking. PLGA/BG (A3) and PCL/HA (A2) display high performance volatility, achieving competitive scores only when mechanical criteria are substantially deprioritized.

To validate the robustness of the PLA/HA top ranking, five distinct weighting scenarios were defined, each representing a different expert priority profile: (i) Baseline: weights as derived from Fuzzy AHP (Table 5); (ii) Biological Focus: Cell Viability and Degradation Rate weights doubled, mechanical criteria weights halved; (iii) Mechanical Focus: Compressive Strength and Elastic Modulus weights doubled, biological criteria weights halved; (iv) Equal Weights: all six criteria assigned equal weight (16.7%); and (v) Cost Focus: Manufacturing Cost weight increased to 40%, remaining weight distributed proportionally. The CCᵢ scores were recomputed under each scenario and are illustrated in Figure 6.

As shown in Figure 6, PLA/HA retained the highest CCᵢ score in all five scenarios, including under the Mechanical Focus weighting where PLA/CNT’s CCᵢ increased substantially. This confirms that the PLA/HA selection is not an artifact of any particular weighting configuration, but reflects genuine multi-criteria superiority. The high sensitivity of PLGA/BG (A3) and PCL/HA (A2) across scenarios corroborates their sub-optimal classification for load-bearing applications: their competitive performance under biologically focused weightings highlights their suitability for non-load-bearing BTE applications (e.g., calvaria repair or soft tissue scaffolding), but confirms their inadequacy for cortical bone defect repair.

## 4. Discussion

### 4.1. The Multi-Criteria Optimality of PLA/HA: A Mechanistic Interpretation

The identification of PLA/HA (A1) as the optimal composite for load-bearing BTE is consistent with its longstanding status as the de facto reference material in the field [21], but the present framework contributes meaningful quantitative specificity that qualitative reviews cannot provide. The “Goldilocks” effect observed in the ranking whereby PLA/HA achieves moderate-to-good performance across all criteria without significant performance limitations on any axis is precisely the outcome that Fuzzy TOPSIS is designed to identify. Its compressive strength TFN of (20, 45, 65 MPa) overlaps effectively with the lower cortical bone range and, critically, with trabecular bone requirements (2–12 MPa) when porosity is controlled, making it versatile across bone types and defect locations [21]. The HA filler plays a dual role that the crisp-value models would not fully capture: it contributes both to mechanical stiffness (raising the elastic modulus above the PLA matrix baseline) and to biological performance, as HA’s chemical similarity to native bone mineral promotes direct osteoblast adhesion and proliferation, while its alkaline dissolution products neutralize the acidic by-products of PLA hydrolysis (lactic acid), thereby improving long-term biocompatibility and reducing local inflammatory response [21].

A key methodological insight concerns the comparison with non-fuzzy ranking results. Under a crisp-value MCDM analysis using midpoint property values, PLA/CNT would likely have ranked first owing to its superior modal compressive strength (47 MPa modal) and modulus (1.7 GPa modal). However, the Fuzzy TOPSIS framework penalized PLA/CNT by incorporating the wide biological uncertainty interval in its Cell Viability TFN (75, 85, 90%), reflecting the documented concentration-dependent cytotoxicity of multi-walled carbon nanotubes reported in recent studies [30]. This “dampening” of mechanical superiority by biological risk is precisely the clinically conservative behavior intended in the framework design: it correctly prioritizes a material with a well-established, predictable safety profile (PLA/HA) over one with superior mean mechanical values but uncertain biological behavior (PLA/CNT).

### 4.2. Critical Analysis of Sub-Optimal Candidates

PCL/HA (A2) ranked fourth (CCᵢ = 0.479) despite strong biological scores. The model correctly identified its fundamental disqualifying characteristic: a yield strength routinely below 1 MPa in composite form, which is mechanically incompatible with primary load-bearing requirements regardless of its superior ductility [22]. This finding underscores a critical distinction that is frequently obscured in qualitative descriptions of PCL composites: high elongation at break and high ductility are not equivalent to load-bearing capacity, and the Fuzzy TOPSIS framework correctly penalizes the former in the context of cortical bone defect repair.

PLGA/BG (A3) ranked last (CCᵢ = 0.452), primarily due to its rapid degradation kinetics: the TFN (4, 8, 12 weeks) reflects a temporal profile incompatible with the 3–6-month window required for adequate cortical bone regeneration [26]. Premature scaffold degradation before sufficient bone matrix deposition would leave the defect site unprotected during the critical early remodeling phase, precipitating mechanical failure. This finding should not be interpreted as a general condemnation of PLGA/BG: it is a context-specific conclusion. For non-load-bearing craniofacial or maxillofacial applications where faster resorption is advantageous, PLGA/BG’s high bioactivity and tunable degradation rate would likely render it a top-ranked candidate.

PLA/Mg (A5, CCᵢ = 0.510) occupied an intermediate third position. Its relatively wide uncertainty interval in degradation rate (12–24 weeks TFN span) reflects the inherent biological unpredictability of magnesium corrosion in physiological environments: the rate of hydrogen gas evolution is highly sensitive to local pH, ion concentration, and implant geometry, and cannot be precisely controlled in clinical conditions [28]. While PLA/Mg holds considerable promise as a next-generation BTE material particularly given recent advances in magnesium alloy compositions and surface coatings the present analysis correctly classifies it as “suitable” rather than “optimal” pending further biological characterization.

### 4.3. Implications of the Fuzzy Approach for Material Selection Practice

The comparison between fuzzy and crisp ranking outcomes has direct methodological implications for how the BTE community should approach computational material selection. The present analysis demonstrates that fuzzy modeling changes the top-ranked material from PLA/CNT (crisp analysis) to PLA/HA (fuzzy analysis) a clinically significant difference. This inversion occurs because the crisp analysis evaluates only the most likely performance value (the modal TFN value m), while the Fuzzy TOPSIS framework evaluates the entire range of plausible values including worst-case scenarios (l). In clinical biomaterials selection, where a scaffold failure could result in re-operation, infection, or permanent disability, designing to worst-case biological performance bounds is the appropriate engineering philosophy [16]. The Fuzzy AHP-TOPSIS framework formalizes this philosophy within a quantitative, transparent, and reproducible computational structure.

### 4.4. Comparison with Existing Literature and Published MCDM Studies

Our finding that PLA/HA is the most favorable candidate for load-bearing cortical scaffolds aligns with the broad consensus in qualitative reviews [14] and previous MCDM studies using hybrid AHP-TOPSIS [31], which identify ceramic-reinforced PLA as a high-performing composite class [31,32]. However, this study provides a unique contribution by demonstrating a ‘ranking inversion’ compared to traditional models. While ‘crisp’ analysis often ranks PLA/CNT first due to its peak mechanical strength, our fuzzy framework demotes it when biological uncertainty is factored in. This reflects a clinically conservative ‘safety-first’ approach that is often missing in purely performance-driven engineering studies. Furthermore, the model accurately captured the limitations of PLGA/BG for load-bearing sites, consistent with recent findings regarding its rapid resorption kinetics [26,33]. Finally, as noted by Jahan and Edwards [29], the choice of normalization is critical in MCDM; our use of TFNs ensures that the final ranking is not merely a reflection of midpoint averages but a robust assessment of the entire property distribution [4,9,29,31].

### 4.5. Limitations

Three limitations warrant acknowledgment. First, the analysis is restricted to five composite candidates and six evaluation criteria; the inclusion of additional alternatives (e.g., 3D-printed gradient scaffolds, strontium-doped HA composites) may alter intermediate rankings. Second, all property values are literature-derived and reflect in vitro testing conditions; in vivo performance may differ due to the complex dynamic mechanical environment and remodeling biology of load-bearing defect sites. Third, the expert panel comprised three members from engineering and surgical backgrounds; expansion to include cell biologists and regulatory specialists may further enrich the criterion weighting.

## 5. Conclusions

This study successfully implemented a hybrid Fuzzy AHP-TOPSIS framework to evaluate five biodegradable composites for load-bearing bone tissue engineering. By utilizing Triangular Fuzzy Numbers (TFNs), the model effectively incorporated the variability and uncertainty inherent in experimental literature. PLA/HA emerged as the most favorable candidate (CCi = 0.677), demonstrating a robust balance between mechanical competence and biological safety that remained consistent across multiple sensitivity scenarios. While nanomaterials like PLA/CNT offer higher peak strength, our fuzzy modeling highlights the clinical risks associated with their biological uncertainty. This computational approach provides a replicable blueprint for evidence-based material selection, potentially reducing the experimental burden in the development of scaffolds for critical-sized bone defects.

## Figures and Tables

**Table 1:** Systematic literature search protocol and study inclusion/exclusion criteria.

Parameter	Specification
Databases	Scopus, Web of Science, PubMed
Search Syntax	(“Polymer Composite” OR “Scaffold”) AND (“Bone Tissue Engineering”) AND (“Mechanical Properties” OR “Compressive Strength”)
Inclusion Criteria	(1) Peer-reviewed research articles published 2021–2025; (2) explicit quantitative reporting of both mechanical and biological properties; (3) focus on porous scaffolds for bone tissue engineering rather than solid bulk specimens.
Exclusion Criteria	(1) Non-biodegradable materials (e.g., titanium, PEEK, stainless steel); (2) studies reporting only qualitative data without numerical property values; (3) review articles (used for contextual background only, not for raw data extraction).
Final Yield	10 peer-reviewed experimental studies providing quantitative data for the five composite candidates.

**Table 2:** Linguistic variables and corresponding Triangular Fuzzy Numbers (TFNs) used for expert judgements in the Fuzzy AHP pairwise comparisons.

Linguistic Variable	TFN (l, m, u)	Definition
Very Low (VL)	(1, 1, 3)	Significantly inferior performance or negligible importance relative to other criteria.
Low (L)	(1, 3, 5)	Below-average performance or marginal importance.
Medium (M)	(3, 5, 7)	Average performance or moderate importance.
High (H)	(5, 7, 9)	Above-average performance or substantial importance.
Very High (VH)	(7, 9, 9)	Superior performance or critical importance; key determinant of clinical success.

**Table 3:** Selected composite candidates and clinical justification for inclusion in the study.

Code	Composite	Clinical Rationale and Justification for Selection
A1	PLA/Hydroxyapatite (PLA/HA)	The benchmark composite. PLA provides a biodegradable, processable matrix; HA mimics the inorganic phase of native bone mineral, promoting direct osteoconductivity. HA also buffers the acidic degradation by-products of PLA, enhancing long-term biocompatibility. Extensively validated in pre-clinical and clinical studies [21,22,23].
A2	PCL/Hydroxyapatite (PCL/HA)	A widely used clinical alternative offering superior ductility and a slower, more controllable degradation profile than PLA. PCL’s low melting point facilitates fabrication at lower temperatures, preserving HA bioactivity [24,25].
A3	PLGA/Bioactive Glass (PLGA/BG)	PLGA allows tunable degradation kinetics by adjusting the PLA:PGA ratio. Bioactive Glass forms a hydroxycarbonate apatite layer on its surface upon contact with physiological fluids, establishing a direct chemical bond with host bone tissue. [26].
A4	PLA/Carbon Nanotubes (PLA/CNT)	Included to evaluate the upper bound of mechanical performance achievable with carbonaceous nano-reinforcement. CNTs provide exceptional tensile strength and stiffness, but introduce cytotoxicity concerns at high concentrations that this study explicitly models via TFNs [27].
A5	PLA/Magnesium (PLA/Mg)	A metal–polymer hybrid included as a comparative model for metallic reinforcement. Magnesium is biocompatible, biodegradable, and contributes to mechanical strength; however, its degradation produces hydrogen gas, introducing biological uncertainty modeled in the fuzzy data matrix [28].

**Table 4:** Fuzzy Master Data Matrix: TFN property values (l, m, u) for all composite candidates across six evaluation criteria.

Code	Material	C1: Strength (MPa)	C2: Modulus (GPa)	C3: Porosity (%)	C4: Degradation (Weeks)	C5: Cell Viability (%)
A1	PLA/HA	(20, 45, 65)	(1.5, 3.5, 6.0)	(40, 60, 70)	(24, 48, 100)	(85, 92, 99)
A2	PCL/HA	(3.5, 7.0, 11)	(0.1, 0.2, 0.4)	(60, 75, 90)	(50, 100, 200)	(90, 95, 99)
A3	PLGA/BG	(2.0, 5.0, 15)	(0.5, 1.0, 3.0)	(80, 90, 95)	(4, 8, 12)	(90, 96, 99)
A4	PLA/CNT	(35, 47, 60)	(1.2, 1.7, 2.5)	(20, 40, 50)	(24, 48, 72)	(75, 85, 90)
A5	PLA/Mg	(15, 30, 52)	(0.8, 1.5, 2.2)	(30, 55, 70)	(12, 16, 24)	(85, 90, 95)

**Table 5:** Fuzzy AHP-derived criterion weights and priority levels for the six evaluation criteria.

Criterion	Criterion Name	Priority Level	Local Weight (TFN)	Global Weight (%)
C1	Compressive Strength (MPa)	Very High	(0.20, 0.25, 0.30)	25.2%
C2	Elastic Modulus (GPa)	High	(0.15, 0.20, 0.25)	18.4%
C3	Porosity (%)	Medium	(0.10, 0.15, 0.20)	12.1%
C4	Degradation Rate (Weeks)	Medium	(0.10, 0.15, 0.20)	12.1%
C5	Cell Viability (%)	High	(0.15, 0.20, 0.25)	21.5%
C6	Manufacturing Cost Index (1–9)	Low	(0.05, 0.05, 0.10)	10.7%

**Table 6:** Fuzzy TOPSIS final ranking of composite candidates, with Closeness Coefficients and performance classifications.

Rank	Composite Candidate	CCᵢ Score	dᵢ (FPIS)	dᵢ^−^ (FNIS)	Classification
1	A1: PLA/Hydroxyapatite	0.677	3.82	8.01	Optimal
2	A4: PLA/Carbon Nanotubes	0.544	5.14	6.10	Suitable—High Strength
3	A5: PLA/Magnesium	0.510	5.49	5.72	Suitable—Balanced
4	A2: PCL/Hydroxyapatite	0.479	5.81	5.33	Sub-optimal—Low Strength
5	A3: PLGA/Bioactive Glass	0.452	6.23	5.12	Sub-optimal—Rapid Degradation

## Data Availability

The complete Fuzzy Master Data Matrix and all computed intermediate values (normalized matrices, ideal solutions, distance calculations) are included within the article. Raw data extracted from individual included studies are available from the corresponding author upon reasonable request.
